# Implementation and Assessment of a Laparotomy-Assisted Three-Port Fetoscopic Spina Bifida Repair Program

**DOI:** 10.3390/jcm12155151

**Published:** 2023-08-07

**Authors:** Corinna Keil, Siegmund Köhler, Benjamin Sass, Maximilian Schulze, Gerald Kalmus, Michael Belfort, Nicolas Schmitt, Daniele Diehl, Alice King, Stefanie Groß, Caitlin D. Sutton, Luc Joyeux, Mirjam Wege, Christopher Nimsky, Wiliam E. Whitehead, Eberhard Uhl, Thierry A. G. M. Huisman, Bernd A. Neubauer, Stefanie Weber, Helmut Hummler, Roland Axt-Fliedner, Ivonne Bedei

**Affiliations:** 1 Department of Prenatal Medicine and Fetal Therapy, Philipps University, 35043 Marburg, Germany; 2 Department of Neurosurgery, Philipps University, 35043 Marburg, Germany; 3 Department of Neuroradiology, Philipps University, 35043 Marburg, Germany; 4 Department of Anesthesiology and Intensive Care Medicine, Philipps University, 35043 Marburg, Germany; 5 Department of Obstetrics and Gynecology, Texas Children’s Hospital, Baylor College of Medicine, Houston, TX 77030, USA; 6 Texas Children’s Fetal Center, Texas Children’s Hospital, Baylor College of Medicine, Houston, TX 77030, USA; 7 Department of Pediatric Neurology, Justus-Liebig University Giessen, 35392 Giessen, Germany; 8 Division of Pediatric Surgery, Department of Surgery, Texas Children’s Hospital, Baylor College of Medicine, Houston, TX 77030, USA; 9 Department of Pediatric Anesthesiology, Perioperative, and Pain Medicine, Texas Children’s Hospital, Baylor College of Medicine, Houston, TX 77030, USA; 10 Division of Neonatology, University Children’s Hospital Marburg, 35043 Marburg, Germany; 11 Department of Neurosurgery, Texas Children’s Hospital, Baylor College of Medicine, Houston, TX 77030, USA; 12 Department of Neurosurgery, Justus-Liebig University, 35390 Giessen, Germany; 13 Edward B. Singleton Department of Radiology, Texas Children’s Hospital, Baylor College of Medicine, Houston, TX 77030, USA; 14 Division of Pediatric Nephrology and Transplantation, University Children’s Hospital Marburg, 35043 Marburg, Germany; 15 Department of Prenatal Medicine and Fetal Therapy, Justus-Liebig University Giessen, 35390 Giessen, Germany

**Keywords:** open spina bifida, fetal therapy, fetoscopic repair, standardized training

## Abstract

Open spina bifida (OSB) is a congenital, non-lethal malformation with multifactorial etiology. Fetal therapy can be offered under certain conditions to parents after accurate prenatal diagnostic and interdisciplinary counseling. Since the advent of prenatal OSB surgery, various modifications of the original surgical techniques have evolved, including laparotomy-assisted fetoscopic repair. After a two-year preparation time, the team at the University of Giessen and Marburg (UKGM) became the first center to provide a three-port, three-layer fetoscopic repair of OSB via a laparotomy-assisted approach in the German-speaking area. We point out that under the guidance of experienced centers and by intensive multidisciplinary preparation and training, a previously described and applied technique could be transferred to a different setting.

## 1. Objective

The incidence of open spina bifida (OSB) is approximately 4.9 per 10,000 live births in Europe and 3.17 in the USA [1,2].

Affected persons typically present with a Chiari 2 malformation and subsequent supratentorial ventriculomegaly, leading to postnatal ventriculo-peritoneal shunt interventions with associated morbidity (e.g., infections, obstructions). Furthermore, they suffer from impaired sensory-motor function of the lower extremities, orthopedic deformities, limitations in bladder and bowel function, and sexual dysfunction depending on the type and level of the lesion [3]. Intrauterine damage to the neuronal OSB structures occurs in two steps [4]. This natural history has been called the “two-hit hypothesis”. The “first hit” is the actual congenital anomaly that results from the lack of closure of the neural tube in the embryonic period. The “second hit” results from direct trauma to the neuronal tissue from fetal movement and from exposure to toxic substances in the amniotic fluid during intrauterine life [5].

The rationale for prenatal therapy is based on the idea of preventing the “second hit” by closing the defect in the second trimester by limiting or reducing the severity of the associated Chiari 2 malformation. The preclinical scientific evidence for this rationale was provided by the experimental work in fetal sheep undertaken by Harrison and colleagues in San Francisco in the 1990s that ultimately culminated in the human randomized controlled trial named the Management of Myelomeningocele Study (MOMS trial) [4]. This trial demonstrated an improvement in motor function and a decrease in hydrocephalus and its shunt rate in children who underwent prenatal closure of OSB. The open surgical approach was a combination of a lower abdominal transverse laparotomy and large hysterotomy at which time the defect was closed using an adaptation of the postnatal neurosurgical technique [6,7,8]. The average gestational age at the time of delivery was 34.1 weeks with 13% delivering before 30 weeks. Delivery via C-section was and remains mandatory with this approach and all further pregnancies [9,10,11].

In an attempt to reduce prematurity and maternal complications, different techniques have been developed. One such technique is a laparotomy-assisted, exteriorized uterus, fetoscopic closure. This has also been called the “hybrid method”. Another minimally invasive technique is the totally percutaneous laparoscopic approach [12,13,14,15]. Similar fetal and infant outcomes have been demonstrated for both techniques as compared to the MOMS trial (preservation of motor function, reduced degree of hydrocephalus). However, there are differences in the preterm birth rate, neonatal outcomes (respiratory distress syndrome and need for NICU management), and the effectiveness of the closure (leakage of cerebrospinal fluid). Later gestational age at delivery was reported for the hybrid technique in comparison to the open and transcutaneous approach that may be attributed to the fixation of the membranes before trocar insertion. Other reasons are less manipulation of the membranes and uterine environment due to the precise placement of the ports directly on the uterus and the gentle hand-held positioning of the fetus. However, these laparoscopic techniques have undergone several modifications making comparison of outcomes difficult [16,17,18,19].

The “International Fetoscopic Myelomeningocele Repair Consortium” was founded in 2017 to compare the results of these laparoscopic approaches. The consortium was an association of international centers with demonstrated expertise in fetoscopic OSB surgery. The aim of the consortium was to evaluate the feto-maternal outcome of the various fetoscopic methods, and in so doing advance the potential for training of new fetal centers by more experienced centers [20,21].

## 2. What Is the Aim of This Current Study?

To report on our experience and then based on the IDEAL recommendations confirm the replication of the safety of this hybrid fetoscopic method, as already reported by other centers [22,23]. Other studies report the outcome of small groups of patients (seven cases, one case) but do not describe the implementation of the surgical approach or the establishment of a multidisciplinary team [24,25]. As part of our training, we were supervised directly by experts from the fetal team of TCH; in addition, essential requirements had to be met prior to the start of the surgery to join the consortium [20].

## 3. Material and Methods

The University Hospital Giessen and Marburg (UKGM) had some experience in prenatal therapy of OSB using the totally percutaneous laparoscopic procedure [26]. Structures and multidisciplinary teams were already in place for the interdisciplinary care of patients. After an in-depth comparison of different techniques, the team decided on the safe and effective “hybrid fetoscopic approach” due to the improved rates of prematurity, fewer maternal risks, and the potential for vaginal delivery [14,20].

In 2019, interdisciplinary preparations for the implementation of the “hybrid procedure” began by gathering different subspecialties involved in the pre- and postnatal care of patients with OSB at the UKGM. Regular meetings took place, followed by the development of treatment concepts in the individual subgroups: diagnostics (neuroradiology, prenatal ultrasound (US), genetics), surgical therapy (gynecology, neurosurgery, prenatal diagnostics, anesthesia), and follow-up care (neonatology, neuropediatric). The surgical team began extensive simulator training as well as surgical staff training [27]. In addition, other disciplines were included in the care structure (pediatric orthopedics, urology and nephrology, obstetrics, and human genetics).

Due to the SARS-CoV-2 pandemic, visits to international training centers for external training following in-house training were not feasible. Therefore, expert exchange and counseling took place through virtual/online platforms.

With online observation of live operations (USA, Spain) and one-to-one exchange between the cooperating disciplines, the preparations were intensified. During the training process, virtual step-by-step exchange and assistance in OSB repair were accompanied by extensive debriefing. Before the first in-vivo OSB repair according to the criteria of the consortium, we fulfilled the full catalog of requirements that was set up to qualify for participation in the “International Fetoscopic Myelomeningocele Repair Consortium” [20].

A clinical trial (prospective cohort study) was approved by institutional review boards in Giessen (161/20) and Marburg (195/20), as well as registration in the German Registry for Clinical Trials (DRKS00026102). After completing the preparations (total time 20 months), the team joined the “International Fetoscopic Myelomeningocele Repair Consortium” in March 2021. The team was supported by centers from the USA (Texas Children Hospital, Houston, TX, USA), Belgium (University Hospital of Leuven, Leuven), and Spain (Clinica Val d’Hebron, Barcelona).

## 4. Results

**Figure 1:**
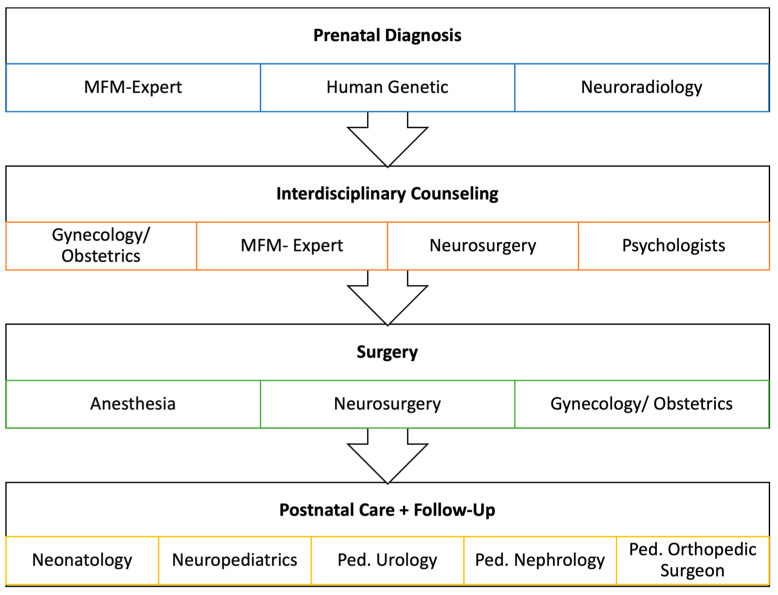
Interdisciplinary team at UKGM: 13 departments are involved in the care of OSB. The center offers the complete spectrum of pre-, peri-, and postnatal care for OSB.

In July 2021, the first case was performed at UKGM in accordance with the criteria of the international consortium and under the guidance of Prof. Michael Belfort, TCH, Baylor College of Medicine (Houston, TX, USA). Since then, a total of 20 patients have been treated at UKGM (as of 07/23). Seven operations were performed with the physical presence of at least one member of the TCH team, with anesthesia, neurosurgery, and neuroradiology guidance from designated experts from the multidisciplinary fetal team of TCH. The “International Fetoscopic Myelomeningocele Repair Consortium” was closed in the summer of 2022 and by then ten cases had been performed at UKGM. Currently, a total of 13 departments at UKGM are involved in the care of OSB (Figure 1). The center offers the complete spectrum of pre-, peri-, and postnatal care for OSB, starting with prenatal diagnosis and therapy with infant follow-up extending to 72 months of life.

**Figure 2:**
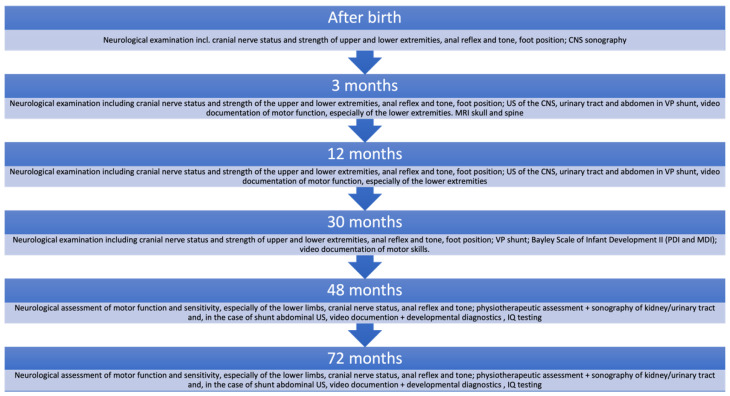
UKGM’s own long-term follow-up: parents are asked to attend regular neuropediatric checkups, MRI imaging is dependent on the child’s clinical condition, but at least once a year. Children are followed up until 72 months of age in a standardized protocol.

We have a standardized protocol for tracking maternal-fetal data. The prospective study records all data collected at each stage of treatment, which are described in detail in the following sections. Figure 2 gives an overview of neuropediatric follow-up as an example.

### 4.1. Diagnostics

Prenatal diagnosis of the fetus includes a detailed fetal anomaly scan, a fetal echocardiogram and central nervous system (CNS)-focused 2D/3D ultrasound, assessment of the anatomic level of the lesion, fetal motor function as described by Carreras [28], evaluation of the posterior fossa and supratentorial structures (e.g., Corpus callosum, ventricular size), and detailed evaluation of any additional CNS anomalies.

OSB nowadays can be detected already in the first trimester. Findings that are suggestive are the “crash sign”, absence of intracranial translucency (IT), and the ratio between brain stem diameter (BS) and its distance to the occipital bone (BSOB) (BS/BSOB ratio). This allows timely presentation in a referral center to further specialized US and MRI to classify the lesion and allow for early fetal therapy already in the second trimester [29,30,31].

Invasive genetic diagnosis including karyotype, chromosomal microarray (CMA), and whole exome sequencing (WES) is offered to all patients. A normal karyotype is mandatory for all patients who pursue fetal therapy at our center.

In terms of prenatal imaging, we regard ultrasound and fetal magnetic resonance imaging (MRI) as complementary modalities. MRI adds important prognostic information for detecting brain anomalies, e.g., abnormalities of the ventricular wall such as heterotopias, and abnormalities of the corpus callosum and the cavum septi pellucidi. Secondly, MRI is useful in confirming our inclusion criteria (e.g., hindbrain herniation), delineates the anatomy, rules out other associated anomalies, and, unlike sonography, is independent of confounding factors such as maternal habitus, fetal position, and bone artifacts [32,33]. Per protocol, hindbrain herniation below the level of the foramen magnum is mandatory for prenatal surgery and must be confirmed on MRI. In addition, MRI adds specific information on any associated findings of Chiari malformation, not accessible on prenatal US (e.g., tectal beaking, cerebellar towering, medullary kinking) as well as further information on anomalies of the corpus callosum [34,35]. Follow-up MRI after in utero repair assesses the integrity of the repair and the potential need for postnatal hydrocephalus treatment [36]. Neuroradiologists should be familiar with either pediatric or fetal MRI. Furthermore, the fetal imaging protocols need subspeciality expertise for the set up. Most standard MRI scanners have very standard and consequently limited fetal imaging protocols. Therefore, optimization on fetal MRI protocol is a continuous process and needs close cooperation by experienced centers and collaboration with the vendors.

### 4.2. Parental Counseling

After completion of all diagnostic steps, parents are counseled by neuropediatric experts regarding the OSB spectrum and resulting limitations and changes in child development. Furthermore, contact with the German support group spina bifida and hydrocephalus (ASBH) is offered [37].

All parents receive the offer of psychological counseling by dedicated psychologists. They provide continuous assistance to the parents from preoperative counseling to neonatal treatment. The low-threshold psychological counseling focuses on reducing anxiety and perceived helplessness through reframing, providing information, and instructing relaxation techniques or using process- and embodiment-focused techniques. Helpful handling of the situation by the entire family, especially the siblings, is discussed or, if necessary, contacts for further help in the home environment are arranged [38].

The surgical team (prenatal diagnostics, neurosurgery, gynecology) consults with the parents regarding the options for OSB management (pre-/postnatal) depending on the feto-maternal conditions. Inclusion criteria for prenatal surgery are based on modified MOMS criteria (see Table 1) [39].

### 4.3. OSB Surgery

Inpatient admission starts 2–3 days prior to the procedure. During this time, further assessments are made by the neonatology, anesthesia, and psychological teams. Preparation for surgery is carried out as described in our standardized protocol including steroid administration (betamethason 2 × 12 mg im.), preoperative prophylactic tocolysis (atosiban and indomethacin), and antibiotics (cefazolin or if allergic clindamycin).

Surgery is performed by a multidisciplinary team consisting of two gynecologists, one neurosurgeon, one fetal medicine specialist (for fetal surveillance), an anesthetic team monitoring the mother and fetus, and a dedicated nursing team and operating room scrub staff.

**Figure 3:**
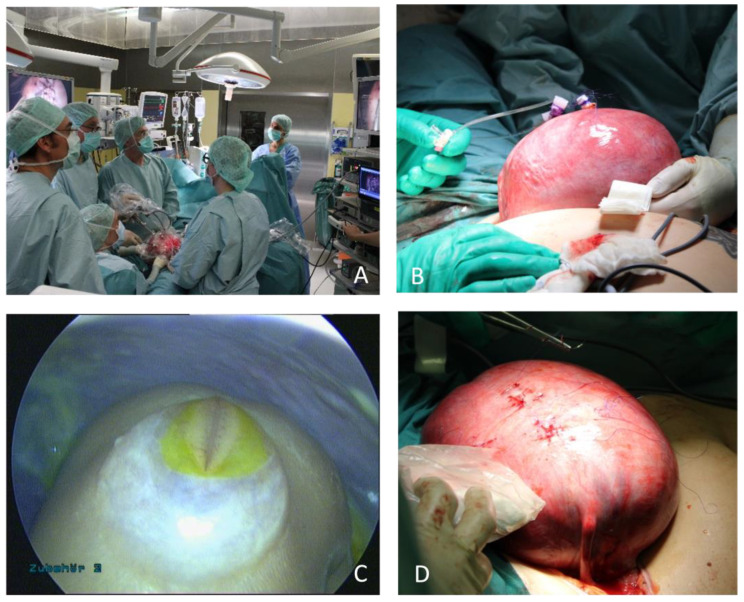
(**A**): Multidisciplinary team during laparotomy-assisted fetoscopic approach (**B**): Exteriorized uterus with 3 ports (**C**): Fetoscopic view on Myelomeningocele (**D**): Uterus after port removal.

First, a low transverse laparotomy is performed, followed by externalization of the uterus. Amniotic membranes are sutured to the uterine wall, and a total of three ports are inserted using the Seldinger technique. Amniotic fluid is removed and replaced with humidified, warmed carbon dioxide. The spinal cord is released sharply, and a bovine Durapatch is applied. After preparing a muscle and skin flap, they are separately closed. After completing suturing, carbon dioxide is removed, and the uterus is refilled with sterile Ringer’s acetate solution. Trocars are removed and defects are closed with transmural sutures. After repositioning the uterus, the maternal abdomen is closed in a standardized way (Figure 3).

The surgery is performed by a gynecologist with special expertise in oncologic and gynecological laparoscopy, a gynecologist/obstetric expert, and a neurosurgeon. Laparotomy and placement and removement of the ports are performed by the GYN/OBS team. Together with the maternal-fetal medicine specialist, they manage the fetal positioning. The maternal-fetal medicine specialist provides fetal surveillance by monitoring umbilical blood flow and heart rate, and, in addition, measures ductus venosus (DV) and arteria cerebri media (a.c.m.) flow at determined timepoints during surgery. The fetoscopic OSB repair is performed under the guidance of the neurosurgeon in collaboration with the gynecological laparoscopic specialist. 

The surgical approach is based on the technique published by the TCH team. The main difference is the three-port instead of the two-port technique [14,40]. Single steps of the repair are presented in Table 2, which also gives an overview of associated problems and possible solutions.

The laparotomy-assisted fetoscopic approach required a realignment of the surgical skills of all team members as well as scrub staff. Through continuous interdisciplinary training, multidisciplinary interaction, enhancement of skills by expert exchange, and external supervision, a multidisciplinary team was created that met the requirements to cover the complex needs of patients with OSB.

The anesthetic management for this surgical procedure consists of a combination of general endotracheal anesthesia administered to the pregnant patient and direct intra-muscular anesthesia administered to the fetus. The approach to anesthesia must account for both maternal and fetal considerations, as both are undergoing distinct surgical interventions, and hemodynamics must be tightly maintained to optimize both maternal and fetal conditions. Any physiologic changes due to anesthetic management (i.e., ventilation, hemodynamic control, and medication administration) impact both unique patients directly or indirectly. To optimize surgical conditions, significant uterine relaxation is required, particularly during phases of direct manipulation of the uterus. Tocolysis is typically achieved using volatile anesthetics combined with other agents (e.g., magnesium sulfate, atosiban, indomethacin). Magnesium sulfate can contribute to postoperative weakness and/or residual neuromuscular blockade when long-acting neuromuscular blocking agents are used, and quantitative neuromuscular blockade monitoring can be useful. Due to the known risk of postoperative pulmonary edema in the context of fetal surgery, a restrictive fluid regimen is used. Both the significant fluid restriction as well as many tocolytic agents can impact maternal and fetal hemodynamic stability, so advanced hemodynamic monitoring is used during surgery and for up to 48 h postoperatively [41,42,43]. A multimodal postoperative analgesic strategy, often including the use of neuraxial analgesia and/or fascial plane blocks, is critical to facilitate early recovery.

The patient receives close postoperative maternal-fetal monitoring, and regular multidisciplinary rounds take place. In an uneventful course, the mother is discharged at day 5–6 after surgery. Ambulatory monitoring is provided weekly by the local OB/Gyn provider and the maternal-fetal medicine specialist at UKGM every 4 weeks. Postoperative follow-up at the center includes sonographic evaluation, including detailed neurosonography, fetal growth evaluation, presence/degree of chorionic amnion separation (CAS), amniotic fluid volume assessment, and measurement of the cervical length. An MRI is performed 6 weeks after the fetal surgery to document regression of hindbrain herniation and quality of the OSB closure. If prenatal inpatient admission is necessary, the patient is ideally transferred to UKGM.

### 4.4. Delivery and Postnatal Care

Delivery should take place in our center, and this is part of the prenatal counseling. In case patients have to travel a long distance, relocation is offered at around 36 weeks if there is no threat of preterm delivery or CAS > 50%. In case of admission to a local hospital, a transfer is organized whenever feasible. In case of emergency (premature rupture of the membranes, threat of preterm delivery), which impedes traveling, patients should present immediately at the closest tertiary care center. UKGM provides guidance and additional information.

At UKGM, we ensure that the choice of delivery mode is based on obstetric indications.

Postnatal care of the newborn is interdisciplinary in terms of neonatology, neurosurgery, and neuropediatrics. Diagnostics include clinical examination, ultrasound, advanced imaging, and the involvement of pediatric orthopedics, urology, and nephrology.

After discharge, the family is referred to a neuropediatric center close to home, as well as to an integrated aftercare model for outpatient treatment for chronically ill patients, which includes various aspects (nursing, social pedagogy, psychology, etc.) and enables central organization and coordination, aiming for close-to-home connections to specialized institutions and seamless transitions between inpatient and outpatient care.

As part of the center’s own follow-up, parents are asked to attend regular neuropediatric checkups, MRI imaging is dependent on the child’s clinical condition but is performed at least once a year. Children are followed up until 72 months of age in a standardized protocol (Figure 2). We plan to expand follow-up through adolescence and into adulthood. The cost of the surgery is EUR 12,050 and is covered by the German National Health Service. Diagnostic and follow-up costs are also covered; therefore, the procedure is accessible to all families.

## 5. Discussion

The implementation of a fetal surgery program for myelomeningocele repair required multidisciplinary collaboration in the setting of a comprehensive program for the care of patients with OSB at UKGM. One challenge we faced internally was in connecting several subspecialties that otherwise have few points of interaction with each other. This required a rethinking of previous treatment concepts.

Structured training by experienced centers played a crucial role in supporting this fetoscopic program and breaking new ground. Joyeux et al. were able to show that the “learning curve” takes longer with the hybrid technique than with standard hysterotomy, which underscores the need for structured training by experienced centers. This study shows that standardized and structured training with the help and supervision of experienced centers is an appropriate and feasible method for supporting a new center in establishing this surgical procedure [17].

A new surgical innovation must be feasible, effective, and safe and needs continuous evaluation. Referring to the IDEAL framework recommendations, the implementation of the laparotomy-assisted three-port, three-layer fetoscopic repair of OSB fulfills IDEAL Stage 2a. Hereby, a new procedure is performed in a single center under close monitoring and data recruitment [22].

It is essential that full transparency with appropriate oversight exist in any such program, regardless of whether it is a new program or an experienced one. Fetal surgery is not yet a well-regulated field worldwide and in order to ensure the highest level of quality, safety, and patient care the highest ethical standards must be maintained. In this, we have made a commitment to our patients and to our colleagues (national and international) to collect and publish the short-, medium- and long-term outcomes of all children and mothers who receive antenatal surgery at UKGM under our standardized protocol. The manuscript of the results of the first 20 cases performed at UKGM is currently in preparation.

The successful establishment of prenatal OSB surgery at the UKGM is the result of a careful strategic and tactical plan, set up and implemented over years, which was driven by the desire to improve the maternal and fetal outcomes of patients with OSB. Using an intense interdisciplinary exchange, a willingness to learn from each other, and the help of external expert coaching, is reflected in the current program at UKGM. In the future, we are looking forward to continuing the interdisciplinary expert exchange and we are committed to the further development and enhancement of the long-term follow-up of our patients in order to improve patient care.

## Figures and Tables

**Table 1:** Inclusion and exclusion criteria according to the modified MOMS criteria.

Inclusion Criteria	Exclusion Criteria
Maternal age > 18 years Singleton pregnancy Myelomeningocele/Myeloschisis at level T1 through S1 Evidence of hindbrain herniation in fetal MRI Gestational age of 19–26 + 0 weeks at surgery Normal karyotype	A fetal anomaly unrelated to OSB Severe kyphosis >/= 30° Obesity, Body mass index (kg/m^2^) > 40 Placenta previa or placental abruption Risk of preterm birth (including short cervix (<20 mm, previous preterm birth) Current or planned cervical cerclage or history of cervical weakness Previous spontaneous singleton delivery prior to 37 weeks Maternal hypertension that would increase the risk of pre-eclampsia or preterm delivery Chronic hypertension with end organ damage and new onset hypertension in current pregnancy Maternal-fetal Rh isoimmunization, Kell sensitization History of neonatal alloimmune thrombocytopenia Maternal HIV or hepatitis-B status positive, known hepatitis-C positivity (screening is not required) Uterine anomaly (large or multiple fibroids, mullerian duct abnormality) Patient does not have social support person Inability to comply with the travel and follow-up requirements of the trial Patient does not meet other psychosocial criteria

**Table 2:** Systematic illustration of the laparotomy-assisted fetoscopic approach for prenatal OSB repair. For each step, the associated problems and possible solutions are described in detail.

Surgical Step	Problem	Solution
Low transverse laparotomy, externalisation of the uterus	Laparotomy is too small, which can lead to compression of the uterine artery, affecting feto-placental perfusion	Extend laparotomy
Placing 3 Ports (12 French, 10 French)	Incorrect port placement will result in difficulty in visualising and accessing the fetal OSB and may compromise surgical closure.	Careful port placement considering placenta, fetal position and operative environment.
	Alteration of placenta/fetus/myometral vessels	Careful sonographic visualisation of (intra-)uterine structures (placenta, fetus, vessels)
Removing amniotic fluid	Can lead to fetal heart alteration	Careful monitoring of fetal heart rate, slow remove
Uterine carbon dioxide insufflation (10 ± 2 mm Hg)	Gas needs to be humidified and warmed, otherwise there is an increased risk of premature rupture of the membranes.	Additional Intermittent irrigation of membranes with sterile solution during OSB repair
	Moderate uterine pressure and moisturisation prevents fetal acidaemia	
Fetal positioning	Can lead to fetal heart alteration, lack of positioning can complicate surgery or make it impossible.	Immediate cessation of fetal manipulation, ongoing fetal monitoring
		Intrauterine resuscitation of the fetus by administration of epinephrine (0.01 mg/kg)
Fetal anesthesia	Can lead to fetal heart alteration, fetal death caused by inccorect dose/application	Assesment of estimated preoperative fetal weight by experienced investigator
(fentanyl 10 ug/kg, cis-atracurium 0.6 mg/kg, atropine 0.03 mg/kg)- every 60 min during OSB repair		Monitoring and administration of fetal anaesthesia using four-eyes principle (MFM and anesthesia)
		Subcutaneous administration under direct vision only
Releasing spinal cord	Too close to skin	May develop inclusion cysts causing severe functional deterioration
	Excessive distance to skin	Injury to the neuronal structures
Preparation muscle flap	Not enough muscle mobilised/present	Close as much muscle as possible
Preparation skin flap	Not enough skin mobilised/present	Relaxing skin incisions lateral of the defect
Placing bovine dura patch	Patch too small/wide/big	Patch preparation according to defect size from MRI/US
Single sutures muscle	Low proportion of muscle	Close as much muscle as possible
Continous closure of the skin	Thin/fragile skin	Relaxing skin incisions lateral of the defect
End of CO_2_-insufflation and instillation of sterile solution	Too much gas/too less sterile solution	Sonographic monitoring of refill
Removal of 3 ports and closure of the myometrial defect	Suture of fetal structures during closure	Controlled suture under sonographic guidance
	Insufficient closure	Uterine loss of amniotic fluid

## Data Availability

Data available on request because of privacy/ethical restrictions.
